# Extending the Generalised Pareto Distribution for Novelty Detection in High-Dimensional Spaces

**DOI:** 10.1007/s11265-013-0835-2

**Published:** 2013-08-16

**Authors:** David A. Clifton, Lei Clifton, Samuel Hugueny, Lionel Tarassenko

**Affiliations:** Institute of Biomedical Engineering, Department of Engineering Science, University of Oxford, Old Road Campus, Roosevelt Drive, Oxford, OX3 7DQ UK

**Keywords:** Novelty detection, Extreme value theory, Patient monitoring, Biomedical engineering

## Abstract

Novelty detection involves the construction of a “model of normality”, and then classifies test data as being either “normal” or “abnormal” with respect to that model. For this reason, it is often termed one-class classification. The approach is suitable for cases in which examples of “normal” behaviour are commonly available, but in which cases of “abnormal” data are comparatively rare. When performing novelty detection, we are typically most interested in the tails of the normal model, because it is in these tails that a decision boundary between “normal” and “abnormal” areas of data space usually lies. Extreme value statistics provides an appropriate theoretical framework for modelling the tails of univariate (or low-dimensional) distributions, using the generalised Pareto distribution (GPD), which can be demonstrated to be the limiting distribution for data occurring within the tails of most practically-encountered probability distributions. This paper provides an extension of the GPD, allowing the modelling of probability distributions of arbitrarily high dimension, such as occurs when using complex, multimodel, multivariate distributions for performing novelty detection in most real-life cases. We demonstrate our extension to the GPD using examples from patient physiological monitoring, in which we have acquired data from hospital patients in large clinical studies of high-acuity wards, and in which we wish to determine “abnormal” patient data, such that early warning of patient physiological deterioration may be provided.

## Introduction

Novelty detection has become a popular method for performing inference in datasets acquired from systems in which there are many examples of “normal” behaviour, but relatively few examples of “abnormal” behaviour. This situation typically arises in condition monitoring, in which we wish to determine the “health” of a complex system, such as a jet engine or a human patient. The novelty detection approach can also be appropriate when the “abnormal” modes of behaviour are poorly-understood, as often arises when there is significant variability between systems; for example, jet engines of the same class may have very different vibration profiles (with “rough” and “smooth” engines), while human patient physiology can vary according to age, demographic background, genetic factors, etc. In such cases, it can be productive to model the “normal” behaviour of a system (sometimes based on data acquired across a population of example systems of the same type), and then detect deviations from normal behaviour.

It is for this reason that novelty detection is sometimes termed *one-class classification*, where there is no explicit model for “abnormal” modes of behaviour because they are too poorly understood, and may vary significantly between systems. Novelty detection, where we wish to identify “novel” examples that have not been seen before (i.e., abnormalities), shares many similarities with *anomaly detection*; in the latter, we similarly wish to detect “abnormal” data, but these may not necessarily be entirely novel - a training set, from which a “normal” model is to be constructed, may contain outliers and other anomalies.

Classification tasks can be separated into generative or a discriminative approaches. In the former we can identify probabilistic structures for the *K* classes $\{C_{i}\}_{i = 1}^{K}$, which may be modelled with pdfs *f* (**x**|*C*_*i*_), **x** ∈ ℝ^*n*^ over the *n*-dimensional data space, and from which we may then define a decision boundary on the level sets of *f *. It is common in novelty detection, for example, to classify test data **x** as being “abnormal” if *f* (**x**|*C*_0_) < *κ*, for some pre-defined threshold *κ*, and where the model of normality^1^ is referred to as *C*_0_. Popular examples of generative methods include the use of finite or infinite mixture models [2, 9, 28] or hidden Markov models [17]. These approaches use most of the training data in an attempt to determine its underlying structure, assuming, as their name suggests, that the data were *generated* from that structure.

In the discriminative approach, it is conventionally argued that the aim of novelty detection is to detect abnormalities, and that therefore the decision boundary is of fundamental importance: there is no need to estimate the class density *f* (**x**|*C*_0_), and that we attempt “never to solve a problem that is more general than the one we actually need to solve” [26]. Discriminative algorithms model the decision boundary *f* (*C*_0_|**x**) directly, and no generative assumption is made. Examples of algorithms in this class include various formulations of the one-class support vector machine (SVM) [26, 30, 31]. In practice, this often means that the decision boundary is defined in terms of a subset of the training data, comprising those examples that exist in what would (by a generative method) be considered the “tail” of the normal model.

The advantage of the generative approach is that its algorithms are typically probabilistic, because a generative data distribution has been constructed. This brings with it all of the advantages of a probabilistic inference system, including the possibility of coping with noise, artefact, and incompleteness in a principled manner (perhaps by marginalisation, if a Bayesian framework is used). The advantage of the discriminative approach is that fewer assumptions have been made about the data, and that classification accuracy is often higher than can be achieved with generative methods.

### Between Generative and Discriminative

An appealing compromise exists between the generative approach (using all of the training data to discover class distribution structure) and the discriminative approach (using only those data that lie close to the edge of the region of support of the “normal” data) to novelty detection. The so-called *peaks-over-threshold* (POT) approach, described in more detail in the next section, is defined for univariate data, and assumes that there is some underlying generative model from which the training data were generated, but that only those data above some high threshold *u* ∈ ℝ (or below some low threshold) are of particular interest. This is used in financial problems, for example, in which we wish to model the distribution of “extremely large” insurance claims, or in flood prediction, in which we wish to model the distribution of “extremely large” water levels. If the threshold is placed at a sufficiently “extreme” location on the univariate axis, then it has been shown that the distribution of the data beyond that threshold (the “peaks over threshold”) tends towards the generalised Pareto distribution [22].

The work described by this paper extends the GPD to data spaces of arbitrary dimensionality, allowing us to model the distribution of probability distributions describing “normal” data in real-world cases, such as in the “health” monitoring of complex systems. The classical univariate POT formulation is described in Section 2, with our GPD extension then introduced in Section 3. Validation is described in Section 4, with discussion and concluding remarks in Section 5.

## Introduction to Extreme Value Theory

Extreme value theory (EVT) aims to model the distribution of data that are “extreme” in magnitude, such as the incidence of earthquakes of extremely large magnitude or the occurrence of rainfall levels of extremely small magnitude. The interested reader is referred to the standard introductory texts for EVT, which mainly come from the statistics literature [7, 12, 14, 24], but which have also generated attention within the engineering literature [3, 19, 20]. Special cases of EVT are also much beloved in the reliability field, when considering the life expectancy of components and systems [21, 32].

The essence of EVT is to model “extreme” data in an *n*-dimensional data space $\mathbb {X} \in \mathbb {R}^{n}$, where some training data exist in a region of high support which may be estimated by a pdf $f_{\mathbb {X}}:\mathbb {R}^{n}\rightarrow \mathbb {R}^+$. Essentially, this is an attempt to perform principled extrapolation into areas of the data space for which very few examples are available. As noted previously, EVT is limited to (most usually) uni- or bivariate data spaces, with data spaces of small *n* being possibly considered using methods such as those involving estimation of copulae [20].

### The Generalised Extreme Value Distribution

The basis of EVT is the Fisher-Tippett theorem [13], a limit theorem [23] which states that if we have a series of random variables (rvs) $\{X_{i}\}_{i=1}^{m}$ which are i.i.d. according to some non-degenerate distribution function^2^ (df) *F*_*X*_, then their maximum tends to a known form *H*_*Y*_ as *m* → ∞, which is the generalised extreme value distribution (GEV):

1 $$ H_{Y}(y) =\left\{ \begin{array}{ll} \exp\left\{-(1+\xi y)^{-1/\xi} \right\} & \textrm{if } \xi \neq 0\\ \exp\left\{ -\exp(-y) \right\} & \textrm{if } \xi = 0 \end{array}\right. $$

where *y* = (*x* − *c*)/*d* is termed the reduced variate, with location and scale parameters *c* and *d*, respectively, and where *ξ* is a shape parameter. The cases *ξ* < 0, *ξ* = 0, and *ξ* > 0 give the Fréchet, Gumbel, and Weibull distributions for maxima, respectively, which are:

2 $$ \text{Gumbel},\ \ \ H^+_{1}(y) = \exp(-\exp(-y))\\ $$

3 $$\textrm{Fr\'{e}chet},\ \ \ H^+_{2}(y) =\left\{ \begin{array}{ll} 0 & \text{if}\,\,y \leq 0 \\ \exp\left(-y^{-\alpha}\right) & \text{if}\,\,y > 0 \end{array}\right. $$

4 $$\text{Weibull},\ \ \ H^+_{3}(y) =\left\{ \begin{array}{ll} \exp\left(-(-y)^{\alpha} \right) & \text{if}\,\, y \leq 0\\ 1 & \text{if}\,\, y > 0 \end{array}\right. $$

for shape parameter *α* ∈ ℝ^+^. These are the limit distributions for the maxima of exponential, heavy-tailed, and light-tailed distributions, respectively. The Weibull is that distribution typically used to estimate the distribution of component lifetimes in reliability theory [10, 21, 32].

The GEV and its subclasses have been used for novelty detection [4–6, 25], where it was used to set a decision boundary (or *novelty threshold*) on a pdf $f_{\mathbb {X}}$, and which is therefore a method that falls into the category of generative methods described previously.

### Peaks Over Threshold

This paper is concerned with the POT method of EVT, which considers exceedances over (or shortfalls under) some extremal threshold *u* ∈ ℝ^*n*^, where typically *n* = 1 or is some small number of dimensions [11]. Assuming that the maximum of a set of rvs {*Y*_*i*_} is “well-behaved” (formally, its distribution tends towards the GEV (1) in the limit; i.e., it is non-degenerate), then it may be shown [22] that the df of the exceedances *Y*_*i*_ > *u* tends towards a known form, which is the GPD:

5 $$ G^{e}_{\mathbb{Y}}(y) =\left\{ \begin{array}{ll} 1-(1+\xi \frac{y-\nu}{\beta})^{-1/\xi} & \textrm{if } \xi \neq 0\\ 1-\exp(-\frac{y-\nu}{\beta}) & \textrm{if } \xi = 0 \end{array}\right. $$

where *ν*, *β*, and *ξ* are location, scale, and shape parameters, respectively. Using (5), we obtain the probability $P(Y - u\ |\ Y > u) = G^{e}_{\mathbb {Y}}$, which merely states that the exceedances of the r.v. *Y * are GPD in distribution (and where we continue to drop subscripts on distributions for clarity).

**Figure 1:**
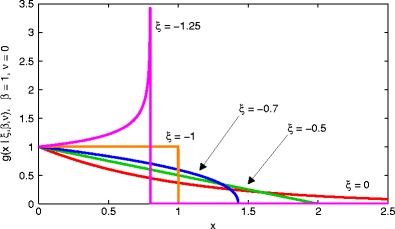
The GPD $g^{e}_{\mathbb {Y}}(x|\xi ,\beta ,\nu )$ for varying *ξ*, with *β* = 1 and *ν* = 0.

The POT method is surprisingly general: as long as our data are non-degenerate in distribution (as is always the case in realistic machine learning and signal processing tasks),they fall into the “domain of attraction” of the GPD. The GPD must, therefore, be a distribution that can take many shapes such that it can be the limiting distribution for exceedances for all non-degenerate distributions. Figure 1 confirms this by showing the pdfs $g^{e}_{\mathbb {Y}}(x|\xi ,\beta ,\nu )$ corresponding to df $G^{e}_{\mathbb {Y}}$ for varying values of the shape parameter *ξ*.

The figure shows that the GPD is flexible according to its shape parameter *ξ*, where we have fixed the values of the location parameter *ν* = 0 and the scale parameter *β* = 1. We can see that the GPD can characterise the distribution of tails where the original pdf $f_{\mathbb {X}}$ is, for example, many of those that we would typically wish to consider in real data analysis problems:

- a heavy-tailed distribution, such as the Student’s *t* distribution (*ξ* > 0);
- the exponential distribution (*ξ* = 0);
- a light-tailed distribution, such as the Gaussian distribution (−0.5 < *ξ* < 0); or
- distributions with compact support (*ξ* ≤ −0.5).

and where the latter set of compact distributions have support given by [0, *β*/*ξ*]. This allows the GPD to represent the tails of the uniform distribution (*ξ* = − 1) and the triangular distribution (*ξ* = −0.5). Monotonically increasing distributions with compact support also fall into the domain of attraction of the GPD, such as the Beta distribution (*ξ* < −1).

The majority of the literature on the GPD is concerned with accurate estimation of the three parameters *ξ*, *β*, and *ν*. It is often the case that the latter can be set to *ν* = 0 if an appropriate normalisation is applied to the data, as will be demonstrated later. Careful consideration must also be given to the value of the threshold *u*, “exceedances” beyond which are those modelled by the GPD. A bias-variance trade-off is apparent in the selection of *u*:

1. If we set *u* to be highly “extreme” (i.e., far from the support of the underlying distribution $f_{\mathbb {X}}$, and hence far into the tails of $f_{\mathbb {X}}$), then the resulting tail data will be very well-fit by the GPD. This is because the GPD is the limiting distribution as |*u*| → ∞, and so, as *u* made more extreme, so the GPD better fits the tail data $\mathbf {X} = \{\mathbf {x} | f_{\mathbb {X}}(\mathbf {x}) > u \}$ in the case of threshold “exceedances” in the upper tail of $f_{\mathbb {X}}$, or $\mathbf {X} = \{\mathbf {x} | f_{\mathbb {X}}(\mathbf {x}) < u \}$ in the case of threshold “shortfalls” in the lower tail.^3^ (For simplicity of description, the remainder of this section will consider a threshold placed in the upper tail of $f_{\mathbb {X}}$, and hence exceedances of *u*). This better fit of the GPD to the tail data corresponds to decreased bias. However, this effect comes at the price of increased variance: there are fewer tail data in set X for more extreme values of *u*, because the threshold is more extreme. This means that any resulting parameter estimates from that small number of tail data are likely to be inaccurate with respect to “true” values, and hence the variance will increase when new observations are considered.
2. Conversely, if we set *u* to be less “extreme” (i.e., close to the support of the underlying distribution $f_{\mathbb {X}}$, and hence not far into the tails of $f_{\mathbb {X}}$), then there will be a large number of exceedances in set X. This will result in less variance, because the resulting parameter estimates will not change as much as if X contained fewer data. However, the price is increased bias, because as *u* becomes less extreme, the GPD will not describe the resulting tail data.

## A Multivariate Extension for the GPD

In most cases of interest that we face in practice, the data cannot be described using univariate distributions, which motivates the multivariate extension to the GPD described in this section.

Conventional statistical approaches to tackling a higher-dimensional dataset would attempt to estimate the dependence structure between univariate marginal distributions. This could be performed using a copula [20], which is a df *C* defined over the dfs of marginal distributions *C* : [0 1]^n^ → [0 1]. That is, we consider our high-dimensional data space to be a unit cube over the *n* marginal distributions, and the copula is that df which takes the *n*-dimensional unit cube as its input. The copula approach is useful in that it fully specifies the dependence between random variables; it is this precision which is, perhaps unsurprisingly, its major limitation, in that it is seldom applied to high-dimensional data spaces.

Alternatives to copulae include estimate the dependence structure between the extremes of each margin (rather than the margins themselves), as is often performed when attempting to extend EVT to bi- or trivariate data spaces [14]. For example, a jet engine condition monitoring dataset could comprise *n* = 50 dimensions [18] or more, where copula estimation or dependence estimation is not feasible.

### The Probability Image Space

We wish to form a GPD over the tails of a distribution $f_{\mathbb {X}}$, where $f_{\mathbb {X}}: \mathbb {X} \rightarrow \mathbb {Y}$ for (potentially highly multivariate) data space $\mathbb {X} \in \mathbb {R}^{n}$, and where $\mathbb {Y}$ is the corresponding probability image space $\mathbb {Y}\in \mathbb {R}^+$; that is, we have the output of the pdf which may take probability densities in the range $\mathbb {Y} \in [0\ y_{\max }]$, for some maximum probability density $y_{\max } = \sup (f_{\mathbb {X}})$. If $f_{\mathbb {X}}$ is a unimodal pdf, then *y*_max_ is simply the modal probability density. We restrict our analysis to non-compact distributions, such as mixtures of exponential kernel functions and other tailed distributions.

In general, $f_{\mathbb {X}}$ may be inconvenient to analyse, as we have made no assumptions about its structure; it could, for example, comprise many modes. The exemplar distribution that we will consider in our patient monitoring case study is a mixture distribution with 400 component distributions, for example. The intuition of our method is to avoid explicitly defining a GPD over the tails of $f_{\mathbb {X}}$ in the *n*-dimensional data space $\mathbb {X}$, but to equivalently define a GPD over the tails of the univariate probability image space $\mathbb {Y}$.

To construct this univariate equivalent form, we must first define a df over the probability image space $\mathbb {Y}$, which we will call $G_{\mathbb {Y}}$:

#### **Definition 1**

$\forall y\in \mathbb {Y}$, let $\text {let}~G_{\mathbb {Y}}(y) = \int _{f_{\mathbb {Y}}^{-1}([0, y])}f_{\mathbb {X}}(\mathbf {x})d\mathbf {x}$.

To understand the definition, we note that this is actually a distribution over level sets on the probability density $f_{\mathbb {X}}$. To assign a value of probability mass $G_{\mathbb {Y}}(y)$ to a level set of probability density value *y*, we integrate $f_{\mathbb {X}}$ between the level set $f_{\mathbb {X}} = y$ and the level set $f_{\mathbb {X}} = 0$. Equivalently, we could say that we are integrating the pdf $f_{\mathbb {X}}$ over all those values **x** in the data space that correspond to probability densities $f_{\mathbb {X}}(\mathbf {x}) \leq y$. This set of all values **x** with $f_{\mathbb {X}}(\mathbf {x}) \leq y$ is the set $f^{-1}_{\mathbb {Y}}\left ([0\ y]\right )$, also known as the pre-image of the set of probability densities [0 *y*]. That is, it is the set of values **x** in the data space which have corresponding densities [0 *y*].

Here we have used the inverse function $f^{-1}_{\mathbb {Y}}:\mathbb {Y}\rightarrow \mathbb {X}^{n}$, which is injective and surjective, but not bijective because many points **x** in data space $\mathbb {X}$ can take the same value of probability density $y\in \mathbb {Y}$. This suggests that while closed forms for $G_{\mathbb {Y}}$ may exist under certain circumstances (unimodality and radial symmetry, to be discussed later), they will not exist in the general case.

**Figure 2:**
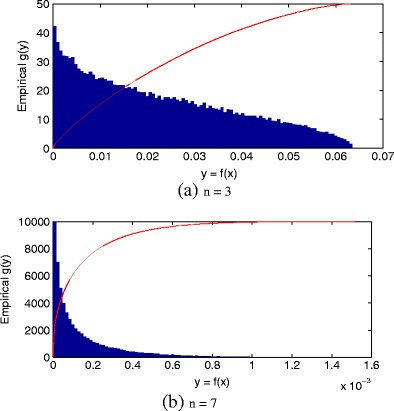
(Unnormalised) empirical pdf $g_{\mathbb {Y}}(y)$ (shown in *blue*) over probability densities *y* = *f* (*x*) when *f * is a standard Gaussian distribution of dimension (**a**) *n* = 3 and (**b**) *n* = 7, when a set of *N* = 10^5^ data were randomly generated from *f *. The corresponding empirical distribution $G_{\mathbb {Y}}(y)$ (shown in *red*) is given for comparison. Note that, for clarity, the dfs $G_{\mathbb {Y}}(y): \mathbb {Y} \rightarrow [0\ 1]$ have been scaled to occupy the same range on the vertical axis as the empirical pdfs $g_{\mathbb {Y}}(y): \mathbb {Y} \rightarrow \mathbb {R}^+$.

A typical df $G_{\mathbb {Y}}$ will take values $G_{\mathbb {Y}}(y)=0$ when *y* = 0, and then increase to $G_{\mathbb {Y}}(y) = 1$ as *y* increases from *y* = 0 to its maximum value, *y* = *y*_max_. Figure 2 shows examples for the cases in which *f * is the standard Gaussian distribution with *n* = 3 and *n* = 7 dimensions, and where we have performed numerical experiments by generating samples from *f *. It may be seen that the empirical df $G_{\mathbb {Y}}(y)$ increases as *y* increases to *y*_max_. It may also be seen that, as dimensionality *n* increases, the value of *y*_max_ decreases from *y*_max_ = (2*π*)^−1/3^ = 0.0635 when *n* = 3 to *y*_max_ = (2*π*)^−1/7^ = 0.0016 when *n* = 7. The (unnormalised) empirical distributions are shown in the figures, where it may be seen that increasing dimensionality causes samples generated from *f * to take decreasing probability density values *y*. This latter effect is expected, due to the fact that the pdf must integrate to unit volume; as the dimensionality of the data space increases, the unit (hyper)volume under the pdf must be spread over those larger dimensionalities of space, resulting in lower overall densities.

Given the above definition of $G_{\mathbb {Y}}$, we may see that another interpretation is that $G_{\mathbb {Y}}(y)$ is the probability of a single random sample $\mathbf {x}$ generated from $f_{\mathbb {X}}$ having a probability density $f_{\mathbb {X}}(\mathbf {x}) \leq y$. As *y* increases from 0 to the modal value $y_{\max }$, then the probability of generating a sample with a lower value of probability density increases. When *y* = *y*_max_, then a random sample generated from $f_{\mathbb {X}}$ will definitely have a lower (or equal) probability density to the modal value $y_{\max }$, and so $G_{\mathbb {Y}}(y_{\max }) = 1$.

### A GPD in the Probability Image Space

We may now use the distribution $G_{\mathbb {Y}}$ over the probability image space $\mathbb {Y}$ to examine the tail behaviour of the underlying pdf $f_{\mathbb {X}}$, which is our goal. We are interested in points **x** in the data space $\mathbb {X}$ which are in some sense “extreme”, and where we will define the notion of “extreme” in terms of the pdf $f_{\mathbb {X}}$.

We can use the POT convergence theorem of [22],

#### **Definition 2**

Let $u \in \mathbb {Y}$ be a threshold in the probability image space of some df $F_{\mathbb {X}}$, with associated pdf $f_{\mathbb {X}}$. Let $G_{\mathbb {Y}}$ and therefore $g_{\mathbb {Y}}$ be the df and pdf that are defined over the probability image space $\mathbb {Y}$. The tail of $G_{\mathbb {Y}}(y)$ is in the domain of attraction of the GPD $G^{e}_{\mathbb {Y}}$ given by (5) for *y* ∈ [0 *u*] as *u* → 0.

This definition effectively allows us to treat “extreme” data as being those that are shortfalls beneath threshold *u* in the probability image space, which is intuitively appealing: “extreme” data are those points **x** in data space $\mathbb {X}$ that our model $f_{\mathbb {X}}$ considers to be improbable.

We emphasise for clarity that in definition 2, the function $G_{\mathbb {Y}}$ is the df defined over the whole probability image space $\mathbb {Y}$, as shown by the red lines in Fig. 2 by contrast, the function $G^{e}_{\mathbb {Y}}$ is the GPD whose domain of attraction contains the tail of $G_{\mathbb {Y}}$ for values of *y* ≤ *u*, examples of which were shown in Fig. 1.

Definition 2 differs from the conventional definition described earlier, where “extremal” data are those with particularly large or small absolute magnitude. We observe that our definition is a general case of the conventional magnitude-based definition: while data with particularly large or small magnitudes will be assigned low probability density by most dfs $F_{\mathbb {X}}$ (and therefore will be “extreme” according to our definition), we also allow points with non-extreme absolute magnitudes to be classified as being “extreme”, as long as they are sufficiently improbable with respect to $F_{\mathbb {X}}$. For a multimodal df $F_{\mathbb {X}}$, this could mean that points points falling between two distinct modes are deemed “extreme”, because they are highly improbable. Most significantly, our definition permits the use of high-dimensional data spaces $\mathbb {X}$, in which it may not be appropriate to restrict “extreme” data to being only those of particularly large or small magnitude in one or more of their margins. For example, in the case of human physiological monitoring, small increases in patient respiration rate combined with small decreases in blood-oxygen saturation may not be large in magnitude (and hence not classified as “extreme” by conventional EVT), but may nonetheless be extremely improbable with respect to a training set of normal patient physiology. In fact, this combination is typically indicative of failing respiratory function: the respiration rate (and heart rate) often increase to try to counteract a falling blood-oxygen level.

It is definition 2 that allows us to consider the method as combining, in some sense, the motivations for the generative and discriminative methods of novelty detection: we have a probabilistic construction, and can, if desired, use the GPD for generation of synthetic “extremal” data and for determining the confidence in our output; we also are interested only in those data beyond the threshold *u*, and so will estimate the parameters of our GPD using only that subset of the training data which are those extremal, “abnormal” examples available to us.

### Investigation using Synthetic Data

**Figure 3:**
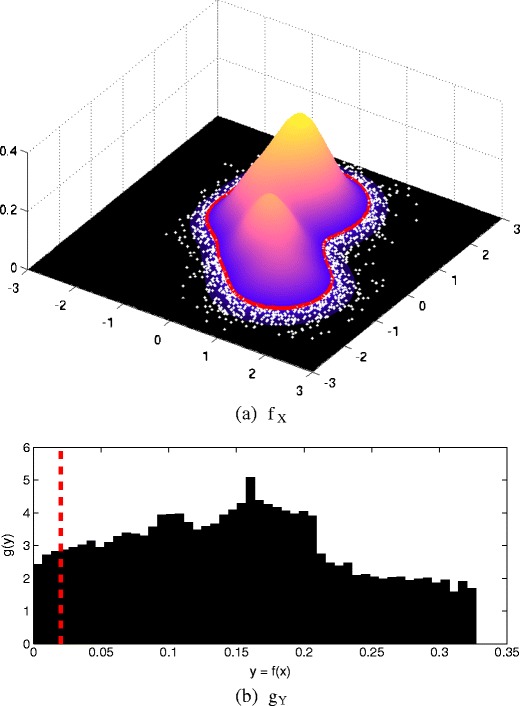
**a** Example bivariate pdf *f*_*X*_, with an extremal threshold *u* (*red line*) and data (*white dots*). **b** Corresponding pdf *g*_*Y*_ over probability image space $\mathbb {Y}$ with threshold *u* (*red line*). Note that the thresholds in both plots correspond.

We now consider the proposed method using an example bivariate pdf $f_{\mathbb {X}}$, shown in Fig. 3a. Here, the distribution may be seen to be multimodal, with two obvious modes. In fact, the pdf is a mixture of eight Gaussian components, each with different covariance matrices.

We then generated *N* = 10^5^ sample points $\{\mathbf {x}_{i}\}_{i = 1}^{N}$ from $f_{\mathbb {X}}$ and determined the corresponding set of probability densities $\{y_{i}\}_{i = 1}^{N} = f_{\mathbb {X}}(\mathbf {x}_{i})$. The empirical distribution $g_{\mathbb {Y}}$ over the {*y*_*i*_} is shown in Fig. 3b, where it may be seen that *y*_max_ = sup{*y*_*i*_} ≈ 0.33, giving the univariate probability image space $\mathbb {Y} \in [0\ 0.33]$ for this example. It may be seen from $g_{\mathbb {Y}}$ that there is a peak occurring at *y* ≈ 0.16, which corresponds to the height (i.e., modal probability density *y*) of the lower of the two modes in the pdf $f_{\mathbb {X}}$ shown in Fig. 3a.

We have selected an example threshold *u* in the probability image space $\mathbb {Y}$ at *u* = 0.015, which is shown by the red line in Fig. 3b. Points **x** with corresponding probability densities $f_{\mathbb {X}}(\mathbf {x})$ falling above *u* are considered to be particularly “normal” points in data space $\mathbb {X}$; conversely, points with $y = f_{\mathbb {X}}(\mathbf {x}) \leq u$ are considered to be “tail data”, and will be used to fit the parameters of a GPD, $G^{e}_{\mathbb {Y}}$.

We emphasise that the “tail data” $\left \{\mathbf {x}:f_{\mathbb {X}}(\mathbf {x})\leq u\right \}$ are not necessarily all to be classified as being “abnormal” data, in the novelty detection sense: they are merely those data who fall in the tail $f_{\mathbb {X}}$ (and hence in the tail of $G_{\mathbb {Y}}$) according to our selection of threshold *u*, and which will, according to definition 2, have a df that is in the domain of attraction of the GPD $G^{e}_{\mathbb {Y}}$ in the univariate probability image space $\mathbb {Y}$. These “tail data” (or “shortfalls beneath adapt the parlence of conventional univariate EVT to our multivariate case) are shown as white points in Fig. 3a, and are found by using the inverse mapping $\{\mathbf {x} | \mathbf {x}\in f^{-1}_{\mathbb {Y}}(y),\ y \leq u\}$.

Having selected a threshold $u \in \mathbb {Y}$, we may now show the same threshold as a level set on $f_{\mathbb {X}}$. This is intuitive: our threshold describes the tails of the pdf in data space $\mathbb {X}$, and so our corresponding GPD over the tail of the univariate probability image space $\mathbb {Y}$ should be expected to form a non-trivial (potentially disjoint) distribution over level sets in the tails of $f_{\mathbb {X}}$ in the data space $\mathbb {X}$.

We now need to estimate the parameters of a GPD using the tail data identified above. By defining a threshold at an example value *u* = 0.015, our GPD will necessarily have compact support $G^{e}_{\mathbb {Y}} \in [0\ 0.015]$, and so, the discussion in Section 2, the shape parameter *ξ* ≤ −0.5. As we saw previously, this subset of GPDs will have compact support [0 *β*/*ξ*], and so *β*/*ξ* = *u* = 0.015. We therefore need only consider fitting fitting *ξ* to our data, and the scale parameter *β* will follow as *β* = *ξu*. Furthermore, the codomain of our GPD is already located at *y* = 0, and so the location parameter of the GPD for our novelty detection detection formulation will be *ν* = 0.

**Figure 4:**
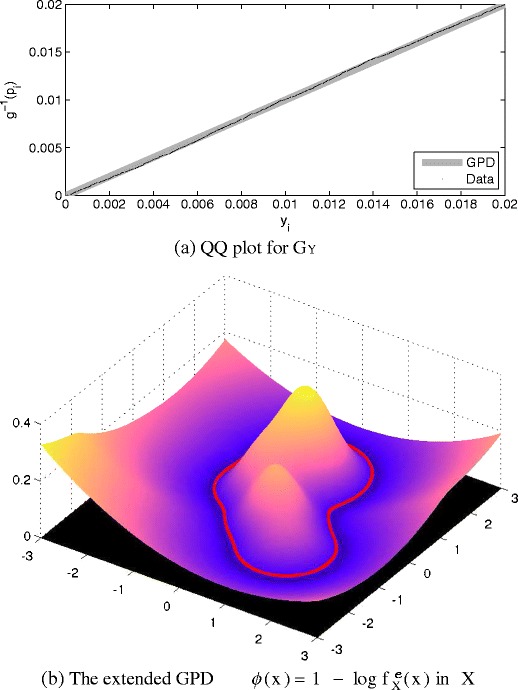
**a** QQ plot comparing the actual tail data {*y* | *y* ≤ *u*} with the inverse of the GPD $(g^{e}_{\mathbb {Y}})^{-1}(p_{i})$ for quantiles $\{p_{i}\}_{i = 1}^{100}$. **b** The extended GPD $\phi (\mathbf {x}) = 1 - \log f_{\mathbb {X}}^{e}(\mathbf {x})$ defined over data space $\mathbb {X}$.

The maximum likelihood method of [3] was used to determine the value of *ξ* from our set of tail data, which has previously been found to be robust for a range of univariate datasets [34, 35]. We may determine the quality-of-fit of the resulting GPD using a quantile-quantile (QQ) plot, as shown in Fig. 4a. Here, the tail data {*y* | *y* ≤ *u*} are plotted on the horizontal axis, while the vertical axis plots the pdf inverse from the GPD $(g^{e}_{\mathbb {Y}})^{-1}(p_{i})$, using percentiles $\{p_{i}\}_{i=1}^{100}$; i.e., percentile *p*_*n*_ = *n*/100. On a QQ plot, a perfectly-fitted model should correspond to the line *y* = *x*, such that predictions from the model (vertical axis) match the training data (horizontal axis), which may be seen to be approximately the case for our example pdf $f_{\mathbb {X}}$.

Finally, with our GPD defined over the univariate probability image space, we can now define an extended GPD in the data space as follows:

#### **Definition 3**

Let $G^{e}_{\mathbb {Y}}(y|\xi ,\beta ,\nu )$ be the GPD that describes the distribution of those data *y* in the probability image space $\mathbb {Y}$ that are shortfalls beneath threshold *u*, such that $G^{e}_{\mathbb {Y}}(y) = P\left (y - u\ |\ \text {abs}(y - u)\right )$. Then we define an extended GPD $f_{\mathbb {X}}^{e}:\mathbb {X}\rightarrow \mathbb {R}^+$ over the data space $\mathbb {X}$ using $f_{\mathbb {X}}^{e}(\mathbf {x}) \propto G^{e}_{\mathbb {Y}}\left ( f_{\mathbb {X}}(\mathbf {x})\right )$.

To determine the constant of proportionality $f_{\mathbb {X}}^{e}(\mathbf {x}) = Z^{-1} G^{e}_{\mathbb {Y}}\left ( f_{\mathbb {X}}(\mathbf {x})\right )$, where Z^−1^ is sometimes termed the partition function, we need to be able to integrate $f_{\mathbb {X}}$ over the the (possibly disconnected) regions of data space $\mathbb {X}$ for which $\{\mathbf {x}\ |\ f_{\mathbb {X}}(\mathbf {x}) \leq u\}$, which is not possible in the case of general $f_{\mathbb {X}}$ but which may be performed for some special cases, such as when $f_{\mathbb {X}}$ is multivariate Gaussian. This is considered in Section 3.4.

An example is shown in Fig. 4b, in which we have performed numerical integration in order to determine Z^−1^ in order to determine the value of $\log f_{\mathbb {X}}^{e}(\mathbf {x})$. The figure shows $\phi (\mathbf {x}) = 1 - \log f_{\mathbb {X}}^{e}(\mathbf {x})$ for the purposes of visualisation, such that regions of data space that are more extreme with respect to the extended GPD take higher values of *ϕ* (**x**). For higher dimensionality *n*, this procedure is impractical, and we recommend that the GPD is used straightforwardly in the probability image space $\mathbb {Y}$, as given in definition 2, rather than mapping it back into the data space $\mathbb {X}$, as given in definition 3. This latter approach will be demonstrated in our case study, in Section 4.

### Closed Forms

For some classes of pdf $f_{\mathbb {X}}$, the distribution in the probability image space $G_{\mathbb {Y}}$ is known in closed form. For example, we have previously shown [4] that for the *n*-dimensional multivariate Gaussian case $f_{\mathbb {X}}(\mathbf {x}) = N(\mathbf {x}\ |\ \mathbf {\mu }, \mathbf {\Sigma })$, $\mathbf {x}\in \mathbb {R}^{n}$ for any $n\in \mathbb {R}^+$, the closed form is

6 $$G_{Y,2p}(y) = y \sum\limits_{k=0}^{p-1} A_{2p}^{k}\left[-2\log(y\,C_{2p})\right]^{(p-k-1)} $$

7 $$\begin{array}{rll} G_{Y,2p+1}(y) &=& y\sum_{k=0}^{p-1}A_{2p+1}^{k}\left[-2\log(y\,C_{2p+1})\right]^{p-k-\frac{1}{2}}\\ &&+\ \text{erfc}{\sqrt{-\log(y\,C_{2p+1})}} \end{array} $$

for all $p\in \mathbb {N}^{*}$, where

8 $$ A_{2p}^{k} = \Omega_{2p}|\mathbf{\Sigma}|^{1/2}\frac{2^{k}(p-1)!}{(p-1-k)!} $$

and

9 $$ A_{2p+1}^{k} = \Omega_{2p+1}|\mathbf{\Sigma}|^{1/2}\frac{(2p-1)! (p-k)!}{2^{k-1} (p-1)! (2p-2k)!}. $$

and where *C*_*n*_ = (2*π*)^*n*/2^|Σ|^1/2^ is the normalising constant (or partition function) and $\Omega _{n}=\frac {(2\pi )^{n/2}}{\Gamma (n/2)}$ is the total solid angle subtended by the unit sphere in $\mathbb {R}^{n}$.

In such cases where the closed form for $G_{\mathbb {Y}}$ is known, the inverse $G^{-1}_{\mathbb {Y}}$ may be used to map the GPD $G^{e}_{\mathbb {Y}}$ in the probability image space back into the data space, to give the extended GPD $f^{e}_{\mathbb {X}}$ in closed form in $\mathbb {X}$. The closed-form solution for the multivariate Gaussian case was derived in derived in [4] because of the analytical convenience of working with the Gaussian, exploiting its radial symmetry; the above equations were specified in terms of the Mahalanobis distance about the mode of effectively making the problem univariate in (Mahalanobis) radius. This approach could similarly be taken for other taken for other unimodal, multivariate probability distributions that are specified radially, which remains future work. There are many algorithms within the field of machine learning for which the Gaussian kernel is used, which makes the above formulation particularly useful.

Proceeding with our extended GPD $G^{e}_{\mathbb {Y}}$ for the case when $f_{\mathbb {X}}$ is multivariate Gaussian, we can define the GPD as a distribution $F^{e}_{\mathbb {X}}$ over contours in the data space $\mathbb {X}$ with closed form:

10 $$F^{e}_{\mathbb{X}}(\mathbf{x}) = 1 - G^{e}_{\mathbb{Y}}\left(y\right)$$

11 $$ = 1 - \left[1 - \left(1 + \frac{\xi}{\beta}y\right)^{-1/\xi} \right] $$

12 $$ = \left(1 + \frac{\xi}{\beta}y\right)^{-1/\xi} $$

13 $$ = \left(1 + \frac{\xi}{\beta C_{n}}\exp\left[-\frac{\mathrm{M}(\mathbf{x})^{2}}{2}\right]\right)^{-1/\xi}$$

where (11) has used the definition of the GPD in (5) with location patameter *ν* = 0, as is the case for our novelty detection formulation. In (13), we have used the radial definition of the multivariate Gaussian *N* (*μ*, Σ), such that M(**x**) = (**x** − *μ*) ^⊤^ Σ ^−1^ (**x** − *μ*) is the Mahalanobis radius of **x** with respect to the *n*-dimensional Gaussian, and *C*_*n*_ = (2*π*)^*n*/2^|Σ|^1/2^ is the Gaussian partition function as before.

## Case Study: Patient Vital-Sign Monitoring

An interesting feature of the conventional (univariate) POT method of EVT is that it models the behaviour of any available tail observations explicitly, rather than extrapolating from a model constructed using the non-extreme “normal” data, as does conventional EVT based on the generalised extreme value distribution (GEV). This is advantageous, because tail observations are comparatively rare with respect to the quantity of non-extreme “normal” data, and thus an estimate of the pdf $f_{\mathbb {X}}$ will be biased towards accurately representing the distribution of the more numerous non-extreme “normal” data. The POT method typically considers exceedances of an extremal *u* which are assumed to occur at times according to a Poisson point process [11]. Therefore, as Therefore, as described in Section 1, the POT method may be informally considered to be a compromise between relying on the entire dataset (as typically occurs with a probabilistic generative method), and relying on those data close to the decision boundary between classes (as typically occurs with a discriminative sparse kernel threshold *u*.

Our proposed multivariate extension to the POT method can therefore benefit from the same advantages, and this section demonstrates the application of our method to novelty detection in large datasets of patient vital signs.

### A Model from Clinical Practice

Previous work has resulted in construction of a pdf $f_{\mathbb {X}}(\mathbf {x}): \mathbb {R}^{4}\rightarrow \mathbb {R}^+$ constructed from a training set of vital sign data (of approximately 3,500 hours in total duration) acquired from 150 “normal”, stable patients in certain high-risk groups of the Oxford University Hospital NHS Trust [29]. This pdf is a mixture obtained using *K* = 400 Gaussian kernel centres, which was then used to assign novelty scores $z(\mathbf {x}) = -\log f_{\mathbb {X}}(\mathbf {x})$ to data. Test data were deemed to be “abnormal” if they exceeded some fixed threshold on the novelty score *z*(**x**) > *κ*_*z*_. The value of the threshold *κ*_*z*_ was determined by minimising the false-positive and false-negative classification rates with respect to previously-unseen “normal” and “abnormal” validation data, respectively.

The resulting system has proven successful in reducing the incidence of physiological deterioration in acutely-ill hospital patients when used to alert clinical staff of “abnormal” patient vital signs during a subsequent clinical trial at the University of Pittsburgh Medical Centre (UPMC) [15, 16]. We will use our multivariate extension to the GPD to explore the tail behaviour of the model $f_{\mathbb {X}}$ with respect to the dataset acquired during the clinical trial at the UPMC.

### Investigating the Extended GPD

The UPMC dataset comprises over 18,000 hours of vital-sign data, collected from 332 high-risk adult patients [15]. The dataset contains measurements of heart rate, respiratory rate, blood-oxygen saturation (acquired at a sampling interval of *τ*_*s*_ ≈ 20 secs) and systolic blood pressure (acquired at *τ*_*s*_ ≈ 30 minutes, which was subsequently up-sampled to the sampling rate of the other three vital signs).

Two subsets of the dataset were identified:

- Set *C*_*a*_, containing periods of patient data deemed to be indicative of “abnormal” patient condition by clinical experts. This set comprises data from 44 patients, of approximately 43 hours in total duration.
- Set *C*_*n*_, containing data from all those patients for whom no “abnormal” event was deemed to have occurred, of approximately 16,000 hours in total duration.

The goal of physiological monitoring is to identify those patients in “abnormal” set *C*_*a*_ as early as possible so that clinicians may be brought to the bedside, while not generating false alarms when presented with data from patients in “normal” set *C*_*n*_.

**Figure 5:**
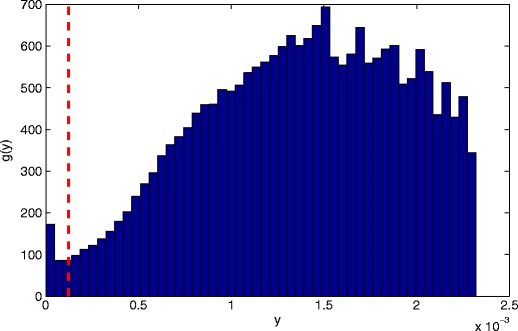
Empirical pdf $g_{\mathbb {Y}}$ over the probability image space for an existing model of normal $f_{\mathbb {X}}$ patient physiology used in clinical practice, obtained using *N* = 10^5^ data generated from $f_{\mathbb {X}}$. A threshold *u* is shown as a *vertical dashed line*, corresponding to $\kappa _{y}$ used as a novelty threshold in clinical practice.

Before formulating our extended GPD for this model $f_{\mathbb {X}}$, we first investigate the probability image space $\mathbb {Y}$, which is shown in Fig. 5. It may be seen that the complex multi-modal pdf results complex behaviour of $g_{\mathbb {Y}}$ in the probability image space, particularly in the range of probability densities corresponding to the most “normal” patient data, *y* > 1.5 × 10^−3^. However, in the tails of see the usual decaying-exponential behaviour that one would expect for the tails of a mixture of Gaussian distributions, observed far from the complex hull formed from their modes. It may be seen that the novelty used in clinical practice at *u* = *κ*_*y*_ falls far into the tail of the distribution, such that the tail is likely is likely to be well-fitted by the GPD.

Following [11], we aim to find the GPD df for the tail data $G^{e}_{\mathbb {Y}}(u - y)$ = $G_{\mathbb {Y}}(u)G^{e}_{\mathbb {Y}}(y)$, which states that the distribution of tail data $G^{e}_{\mathbb {Y}}(u - y)$ is some version of the GPD $G^{e}_{\mathbb {Y}}(y)$ scaled by a factor $G_{\mathbb {Y}}(u)$. A natural estimator for this factor $G_{\mathbb {Y}}(u)$ is given by the empirical df

14 $$ \hat{G}_{\mathbb{Y}}(u) = \frac{1}{N}\sum\limits_{i=1}^{N} I_{\{y_{i}<u\}}=\frac{N_{u}}{N} $$

where *I* is the indicator function, which counts the number *N*_*u*_ of tail data {*y*_*i*_} falling beneath the threshold *u*. This, with (5), leads immediately to the resulting estimator

15 $$ \hat{G_{Y}}(u - y) = \frac{N_{u}}{N}\left(1-\xi\frac{y}{\beta}\right)^{-1/\xi}  $$

In this illustration of our extension of the GPD to multivariate data, we consider the threshold *u* = *κ*_*y*_, where $\kappa _{y} \in \mathbb {Y}$ is that threshold that corresponds to the location in the probability image space of the threshold $\kappa _{z}$ on the original patient vital-sign model $f_{\mathbb {X}}$. That is, all probability density values $\{y \in \mathbb {Y}\ |\ y < \kappa _{y}\}$ are isomorphic to novelty scores *z* (**x**) > *κ*_*z*_, because $\forall \mathbf {x} \in \mathbb {X}$, $z(\mathbf {x}) = z\left (f^{-1}_{\mathbb {Y}}(y)\right )$, allowing us to map from probability densities *y* to novelty scores *z* (**x**). Therefore, because all these points {**x**} exceed the novelty threshold *κ*_*z*_ in the original original algorithm, they would therefore all be classified as being “abnormal” with respect to the model respect to the model therefore represents an “extremal” threshold, and indeed the quantile on $f_{\mathbb {X}}$ (i.e., $\bar {G}_{\mathbb {Y}}$, the survival function of $G_{\mathbb {Y}}$) that corresponds to $\kappa _{z}$ is approximately $\bar {G}_{\mathbb {Y}} = 0.985$, as may be seen in Fig. 5, where we see that there is little probability mass existing in the tail of $g_{\mathbb {Y}}$ below the threshold *u*. This quantile 0.985 is traditionally considered as being sufficiently considered as being sufficiently extreme (i.e., close to 1) for the assumption to hold that the tail distribution lies within the domain of attraction of the GPD [7].

### Fitting the Extended GPD

**Figure 6:**
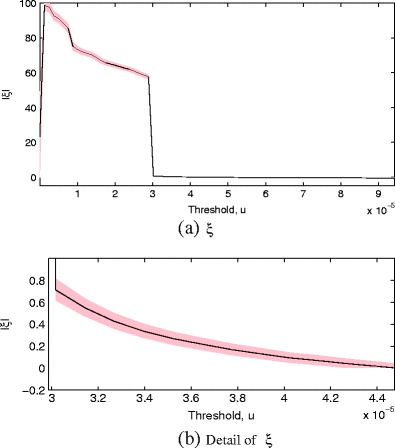
Plots of the maximum likelihood estimate for shape parameter *ξ* with changing threshold *u*, showing (**a**) *ξ* over a large range of thresholds, and (**b**) detail of *ξ* for a small range of values. Note that |*ξ*| has been shown in the plots. 1 standard error in the estimation is shown by the shaded area.

We investigate the variation in maximum likelihood estimates of the shape parameter ξ over a range of threshold values *u*, in keeping with best practice for determining the sensitivity of ξ to choices of *u*. Figure 6a shows |ξ| varying over a range of very small values for *u* > 3 × 10^−5^, where it be seen that the estimate is stable until the threshold is moved into the most extreme tail *u* ≤ 3 × 10^−5^ of of the probability image space $\mathbb {Y}$. This is expected behaviour, because there are fewer extreme data available as the threshold is decreased in $\mathbb {Y}$, and eventually we are modelling a tail so extreme that parameter estimates become highly unreliable, and ξ rapidly takes unstable values as *u* < 3 × 10^−5^. However, we require only a value of ξ that is stable over the majority of the tail region, and Fig. 6 shows that ξ → 0 as *u* increases to the right of the plot.

**Figure 7:**
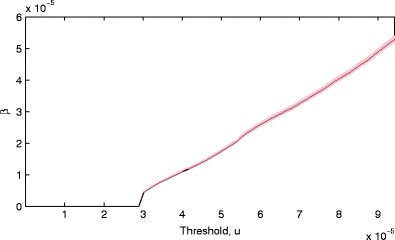
Plots of the maximum likelihood estimate for scale parameter *β* with changing threshold *u*. 1 standard error in the estimation is shown by the shaded area.

A similar investigation for the stability of the scale parameter *β* over a range of threshold values *u* indicates that *β* varies approximately linearly with *u*, as is expected: by increasing *u*, we are directly increasing the support of the GPD $G^{e}_{\mathbb {Y}}$ in the probability image space as [0 *u*] = [0 *β*/ ξ], described previously, and hence *β ∝ u*, as seen in Fig. 7.

**Figure 8:**
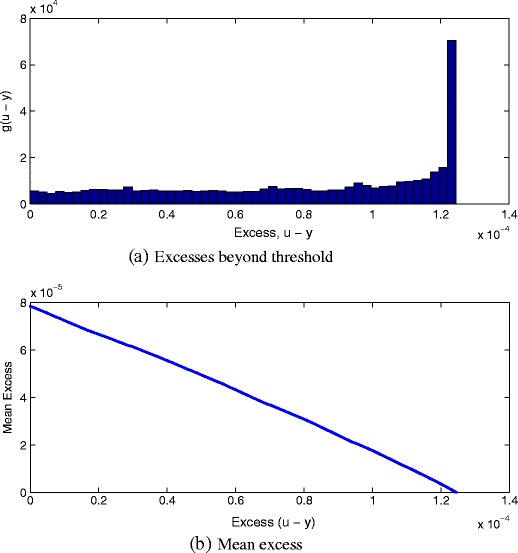
**a** Empirical distribution of excesses (*u* − *y*) beyond threshold *u*. **b** Mean excess plot.

A final check of the suitability of the GPD for modelling the tails of our complex multivariate distribution $f_{\mathbb {X}}$ is to consider the *mean excess*. We define an excess beyond (i.e., below) the threshold to be the quantity *u* − *y*, the empirical distribution of which is shown in Fig. 8a. Here it may be seen that many excesses are small (i.e., close to the threshold *u*), but that there is a significant number of excesses that exist very far from the threshold *u*, shown in the figure as the rightmost peak. It is this peak i the most extremal data that caused the unstable behaviour in values of ξ when *u* was set to be very small, as previously seen in Fig. 6a. In patient physiological data, this peak corresponds to periods of extreme and prolonged physiological derangement [15].

Following [12], we use the mean excess function *e*_*n*_ (*u*) over all *N* data:

16 $$e_{N}(u) = E(y - u\ |\ Y < u)$$

17 $$ = \frac{\Sigma_{i=1}^{N}(y_{i} - u)I_{\{y_{i} < u\}}}{\Sigma_{i=1}^{N} I_{\{y_{i} < u\}}} $$

where *I* is the indicator function, and so *e*_*N*_ (*u*) is the sum of excesses beyond *u*, divided by the number of exceedances. This is the expected value of an exceedance, given that an exceedance has occurred.

If the tail is in the domain of attraction of the GPD, then it may be shown [12] that:

18 $$ e(u) = \frac{\beta + \xi u}{1 - \xi} $$

and hence the mean excesses *e* (*u*) for a truly GPD tail will be linear in *u*. We check the linearity of the mean excesses as shown in Fig. 8b, which shows that the mean excess decreases linearly with excess (*u* − *y*) for our complex model, and hence the GPD is appropriate for modelling the tails.

### Evaluating the Fit

**Figure 9:**
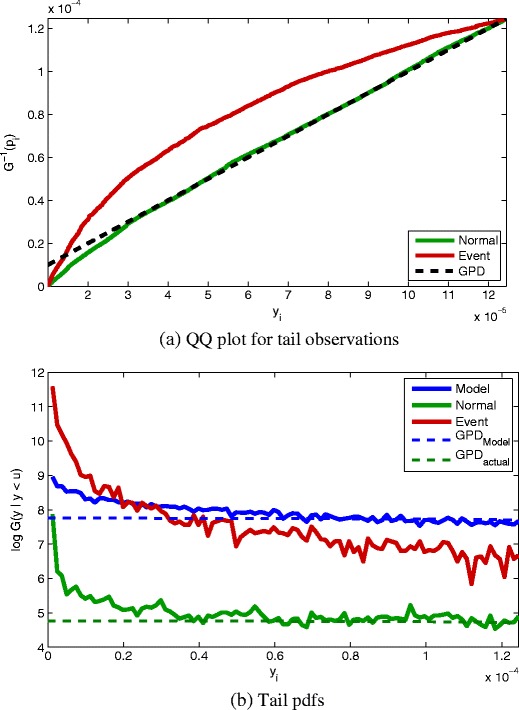
**a** QQ plot showing fit between the GPD (*dashed line*) and extreme-but-normal observations from set *C*_*n*_, with extreme-and-abnormal observations from set *C*_*a*_ shown for comparison. **b** Pdfs of actual extreme-but-normal tail observations (*C*_*n*_), tail observations expected by the model (i.e., synthetic data generated from $f_{\mathbb {X}}$), and extreme-and-abnormal tail observations (*C*_*a*_), with GPDs fitted to the “normal” and model observations (*dashed lines*).

The suitability of this threshold *u* is shown by the fit of the GPD to the “normal” tail observations from *C*_*n*_ in the QQ plot shown in Fig. 9a, where it may be seen that the GPD and the *C*_*n*_ tail observations observations differ only in the left-hand tail of this tail-plot, corresponding to the extremes of this extremal dataset. It is recognised in the literature that the “extremes of the extremal data” will diverge from the distribution given by parameters estimated over the majority of the extremal range [3]. While these extremes of the extremal data will be closely fit by a GPD (they are further into the asymptotic attractor of the GPD as *u* → 0 in this case), the variance of the estimates of the parameters for that GPD increases, as described previously. described previously. Noting that the entire set of tail observations are already extremal with respect to the would all be classified as examples of “abnormal” patient physiology by the original algorithm in [15], with respect to the model $f_{\mathbb {X}}$. Therefore, an alert of the vital-sign monitoring system would occur for every point {**x**}, even though many of these data are extreme-but-normal; i.e., they come from set *C*_*n*_, which contains patients with no physiological deterioration, and for whom any alert of the monitoring system will be a false alert. monitoring system will be a false alert.

The fit of the GPD to the majority of this range, as shown in the figure, is appropriate. We emphasise that while these data are extreme, they are “normal” in that they derive from stable patients in set *C*_*n*_ for whom we wish no alert of the monitoring system to be generated.

Figure 9a also shows that the distribution of the tail observations acquired from unstable, “abnormal” patients in set *C*_*a*_ is significantly different to this GPD, suggesting that we can discriminate between the two cases: the extreme-but-normal data (from *C*_*n*_), and the extreme-and-abnormal data (from *C*_*a*_).

### The Disadvantages of Extrapolation

Figure 9b illustrates why the proposed approach provides an advantage over existing techniques. As noted previously, existing methods [4, 15, 16, 29] assume strong dependence on the model of normality, $f_{\mathbb {X}}$, and typically extrapolate into the tail areas of data space based on $f_{\mathbb {X}}$ (which was constructed using entirely normal data).

The figure shows (in blue) the tail observations corresponding to $f_{\mathbb {X}}$; i.e., the distribution of tail observations $\{y_{i}\}_{i=1}^{N}$ corresponding to a set of generated data $\{x_{i}\}_{i=1}^{N} \sim f_{\mathbb {X}}$, $N = 10^{6}$. If it were appropriate to extrapolate from the model of normality $f_{\mathbb {X}}$ into the tails, then the blue line should closely match the tail model given by the GPD, which is the true limiting distribution in the tail.

However, it may be seen from the figure that these synthetically-generated tail observations (in blue), which are distributed according to the model $f_{\mathbb {X}}$ using (15), are significantly different in distribution to the *actual* tail observations of “normal” patient data, $C_{n}$ (shown in green). The GPD fitted to the tails of the model (shown by the green dashed line) is correspondingly different to the GPD fitted to the actual tail observations of patient data (shown by the blue dashed line). Therefore, we may conclude that (perhaps unsurprisingly) the model of normality $f_{\mathbb {X}}$ does not exhibit the desired tail behaviour; that is, the model of normal patient physiology does not accurately model the tails of the distribution, where physiology tends to be more “abnormal”.

### Advantages of the Extended GPD

The GPD extended to be defined over a probability image space makes the implicit assumption that observed data are i.i.d. with respect to the pdf $f_{\mathbb {X}}$. If the assumption holds, then the exceedances of the extreme threshold *u* follow a Poisson point process [14, 24]. To check the validity of this assumption, we can consider the occurrence of *records* in the dataset.

Adapting the exceedances-over-threshold case from [12] to our shortfalls-under-threshold case in our novelty detection formulation, we define:

#### **Definition 4**

A *record* in our probability image space $\mathbb {Y}$ occurs at point index *i* = 1…*N* if *y*_*i*_ < *m*_*i*−1_ where *m*_*i*−1_ = min (*y*_1_, …, *y*_*i* − 1_ is the minimum of all previous data. We assume that *y*_1_, which is the probability density of the first point in the dataset $y_{1} = f_{\mathbb {X}}(\mathbf {x}_1)$, is the first record.

We use the record-counting process [12] to determine the index *N*_*i*_ at which the *i* ^th^ record occurred:

19 $$ N_{1} = 1$$

20 $$ N_{i} = 1 + \Sigma_{k=2}^{i} I_{\{y_{k} < m_{k-1}\}}$$

It may then be shown [12] that if the data are i.i.d., then

21 $$ E[N_{i}] = \Sigma_{k = 1}^{i}\frac{1}{k} $$

22 $$\text{var}[N_{i}] = \Sigma_{k=1}^{i}\left(\frac{1}{k}-\frac{1}{k^{2}}\right)$$

and that, remarkably,

23 $$ \lim\limits_{i\rightarrow\infty} \left(E[N_{i}] - \log i\right) = \gamma $$

for Euler’s constant *γ* = 0.5772 … We can therefore compare the expected number of records from (21), having variance (22), at index *N*_*i*_ from the counting process of the truly i.i.d. series to the actual observed number of records from the probability image space $\mathbb {Y}$ of our dataset.

**Figure 10:**
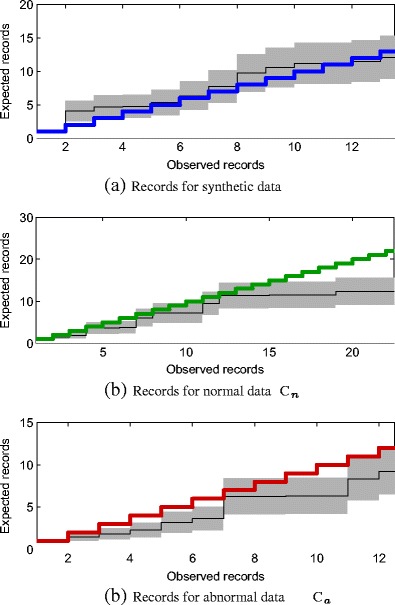
Plots showing actual number of observed records (*horizontal axis*) against the expected occurrence of records (*vertical axis*) after observing the same number of data. For each value on the horizontal axis, the expected number of records (and one standard deviation) are shown by the *thin line* (and shaded background). The *thick line* *y* = *x* is shown in each case, corresponding to the number of actually-observed records occurring at the same time as the expected number of records. Datasets used in each plot are (**a**) *N* = 10^5^ synthetic data generated from (and hence i.i.d. according to) $f_{\mathbb {X}}$, (**b**) normal data, *C*_*n*_, and (**c**) abnormal data, *C*_*a*_.

This is shown in Fig. 10, where we compare the expected number of records from an i.i.d. series on the horizontal axis to the actual observed number of records at that same index *N*_*i*_ in the dataset. Figure 10a shows the case for a set of *N* = 10^5^ synthetic data, generated from $f_{\mathbb {X}}$. By definition, these data are i.i.d. with respect to $f_{\mathbb {X}}$. It may be seen that the occurrence of records in this truly i.i.d. synthetic dataset occur at approximately the place in the dataset that we would expect them to occur: thin line shows the expected number of record at the data index $N_{i}$ where each observed record actually occurred, and occurred, and the thin line (with its variance shown as the shaded background) is close to the line *y* = *x* (shown by the thick line). This indicates that the expectations and actual observations occur at approximately the same rate, as one would hope because the synthetic data are i.i.d. by definition.

Figure 10b shows the case for normal patient data, from set *C*_*n*_. Here, it may be seen that more records are being actually observed than would be expected from a truly i.i.d set of data. For example, when we observe the 20^th^ record (on the horizontal axis) we are expecting only to have seen 12.3 records (±3.3 records). This suggests that the normal patient data contain more extremal data than a truly i.i.d. series.

Figure 10c shows the case for abnormal patient data, from set *C*_*a*_, where it may be seen that the abnormal data are similarly generating more records than would be expected from a truly i.i.d. series. E.g., when the 6^th^ record occurs (on the horizontal axis), we would expect to have seen only 3.6 records (±1.4 records) if the process were i.i.d. In fact, we actually observe 4 records at *N*_*i*_ = 21, quite close to the start of the dataset.

From this we may conclude that the i.i.d. assumption is too strong for our realistic dataset, which is unsurprising: patient data are susceptible to varying dynamical behaviour as the physiological condition of the patient changes. However, despite the fact that the i.i.d. assumption does not hold, it has been shown previously that the GPD, extended over the probability image space $\mathbb {Y}$, is an accurate description of the distribution of tail data - as has been shown both in terms of mean excess, in Fig. 8b, and QQ difference, in Fig. 9a. This suggests that our extended GPD approach is robust to non-i.i.d. behaviour, which is a significant advantage compared with conventional methods that rely solely on the pdf $f_{\mathbb {X}}$ to perform novelty detection. That is, even though the i.i.d. assumption may not hold, and hence the statistics of the data are not accurately described by $f_{\mathbb {X}}$, they have been shown to be accurately described by the extended GPD.

Of particular interest, it may be seen that the extended GPD corresponding to the extreme-but-normal data (shown in green in the figure) differ considerably from that corresponding to the extreme-and-abnormal patient data (shown in red). This result therefore suggests a method of discriminating between tail observations from “normal” patients” and tail observations from truly “abnormal” patients, all of which would have simply been classified “abnormal” by a conventional density-thresholding technique, because all of these observations lie in the tails of the model $f_{\mathbb {X}}$, beyond its threshold *κ*_*z*_.

This effectively offers the potential advantage of being able to push the classification decision beyond the conventional decision threshold *κ*_Z_, and separate the extreme-but-normal from the extreme-and-abnormal.

## Concluding Discussion

We have shown that the tails of an arbitrary pdf $f_{\mathbb {x}}$ tend to the GPD in the probability image space corresponding to the pdf. This results directly in a GPD over the level sets in the pdf $f_{\mathbb {X}}$, defining a complex distribution in the original data space $\mathbb {X}$ which we refer to as the extended GPD.

We have described how our extended GPD can model the tails of pdfs in data space $\mathbb {X} \in \mathbb {R}^{n}$ of any dimension. We assume some model of normality $f_{\mathbb {X}}$ to obtain a mapping into the probability image space $\mathbb {Y}$ corresponding to $f_{\mathbb {X}}$. However, unlike previous approaches, we then directly model any available *actual* tail observations, rather than extrapolating into extremal regions of $\mathbb {R}^{n}$ based on a model of model of normality as has been previously performed.

We directly fit a model to any available extreme-but-normal observations, rather than assuming that the model $f_{\mathbb {X}}$ applies throughout the tail regions. This is advantageous because $f_{\mathbb {X}}$ is biased towards fitting the mass of observations that are “normal” in the training set (assuming that tail observations are less frequently observed than “normal” observations, as is typical in most practical applications). This direct modelling of tail observations is in keeping with the spirit of the univariate approaches in the POT literature, in which the tails are modelled with a GPD while the non-extremal data may be modelled with a different distribution [1, 3, 11].

We have demonstrated that, for a large example dataset of patient vital signs, there is a significant difference between that which the model $f_{\mathbb {X}}$ tells us we *should* expect in the tails (the blue line in Fig. 9b), and the distribution of our *actual* extreme-but-normal tail observations (from set *C*_*n*_). This is to be expected, as it is unlikely that extremal “normal” patient physiology is distributed similarly to the mass of “normal” patient physiology that occurs nearer the modes of $f_{\mathbb {X}}$. This confirms that we should not extrapolate from $f_{\mathbb {X}}$ into its tails, and that we should instead model the tails directly. Our method allows us to represent accurately those extremal data that are available, rather than relying on extrapolation from our model of “normal” physiology.

Furthermore, we have demonstrated the robustness of our method to non-i.i.d. data. Whereas existing methods require that the data are i.i.d. according to the pdf $f_{\mathbb {X}}$ (an assumption that we have shown does not hold for our realistic example), our method based on the extended GPD accurately describes the statistics of the tail data, even when the i.i.d. assumption does not hold (as is the case for most realistic datasets, with non-trivial dynamics).

### Separating Extreme-but-Normal from Extreme-and-Abnormal

In addition, the proposed method allows us to venture out beyond the conventional boundary between “normal” and “abnormal” regions of data space, and to take into the account (using our example of patient vital-sign monitoring) that there is a significant quantity of observations from stable, normal patients beyond that boundary (the extreme-but-normal data), which we would like to separate from observations from truly abnormal patients (the extreme-and-abnormal data), who require clinical attention.

The value of the threshold $u \in \mathbb {Y}$ in our illustration was selected to coincide with an existing decision threshold that is used in clinical practice. The selection of *u* is a selection over a univariate rv, and so any of the standard methods from the literature on univariate EVT for selecting the value of *u* may be employed, such as plots, mean-excess plots, etc. [11] While there is typically a region of $\mathbb {Y}$ in which *u* results in stable estimates of the GPD shape parameter ξ, making such univariate explorative techniques useful, useful, there is scope for Bayesian estimation of this threshold, for which the interested reader is directed to the surveys [8, 34, 35], which are mostly based on non-deterministic approximations, such as those involving MCMC.

### Future Work

We have provided a formulation for a distribution over the level sets in the tails of an arbitrary distribution $f_{\mathbb {X}}$, and have demonstrated the method using a mixture of Gaussian distributions, with parameters obtained using maximum likelihood methods from the EVT literature. Extensions of this work could use the proposed method to model the tails of so-called Bayesian mixture models, in which the parameters of the mixture (including the number *K* of mixture distributions) are set using variational Bayesian methods [2, 27, 33], or other deterministic approximations.

## Footnotes

1 Novelty detection is arguably a hypothesis test, in which the null hypothesis is that test data are drawn from some model of normality, *H*_0_.

2 A degenerate df assigns all probability mass to a single point in the data space.

3 The terms “exceedances” and “shortfalls” are commonly used in the EVT literature.
